# A case of erythema annulare centrifugum cleared with upadacitinib

**DOI:** 10.1016/j.jdcr.2023.09.028

**Published:** 2023-10-08

**Authors:** Valerie Foy, Meagen M. McCusker, Michael J. Payette

**Affiliations:** a Philadelphia College of Osteopathic Medicine, Philadelphia, Pennsylvania; b Integrated Dermatology of Simsbury, Simsbury, Connecticut; c Central Connecticut Dermatology, Cromwell, Connecticut

**Keywords:** erythema annulare centrifugum, JAK inhibitor, rinvoq, upadacitinib

## Introduction

Erythema annulare centrifugum (EAC) is an uncommon skin condition marked by annular, erythematous patches and plaques that often exhibit a classic overlying trailing scale. The pathogenesis of EAC is not fully understood but might be due to a delayed hypersensitivity reaction caused by external or internal stimuli.^1^ To the best of our knowledge, this is the first case report demonstrating the use of upadacitinib for the treatment of EAC.

## Case report

A 65-year-old male presented to the office with a 10-year history of a rash involving the buttocks and lower extremities with less involvement of the upper extremities. He reported a cyclical pattern of eruptions with blistering and subsequent flaking that “moved around” in an advancing pattern. Symptomatically, he noted itching and occasional pain. Stable medications included metoprolol, lisinopril, pravastatin, metformin, fluoxetine, and alprazolam. He is a daily smoker (1/2 packs per day) and consumes 3 alcoholic drinks daily.

**Fig 1:**
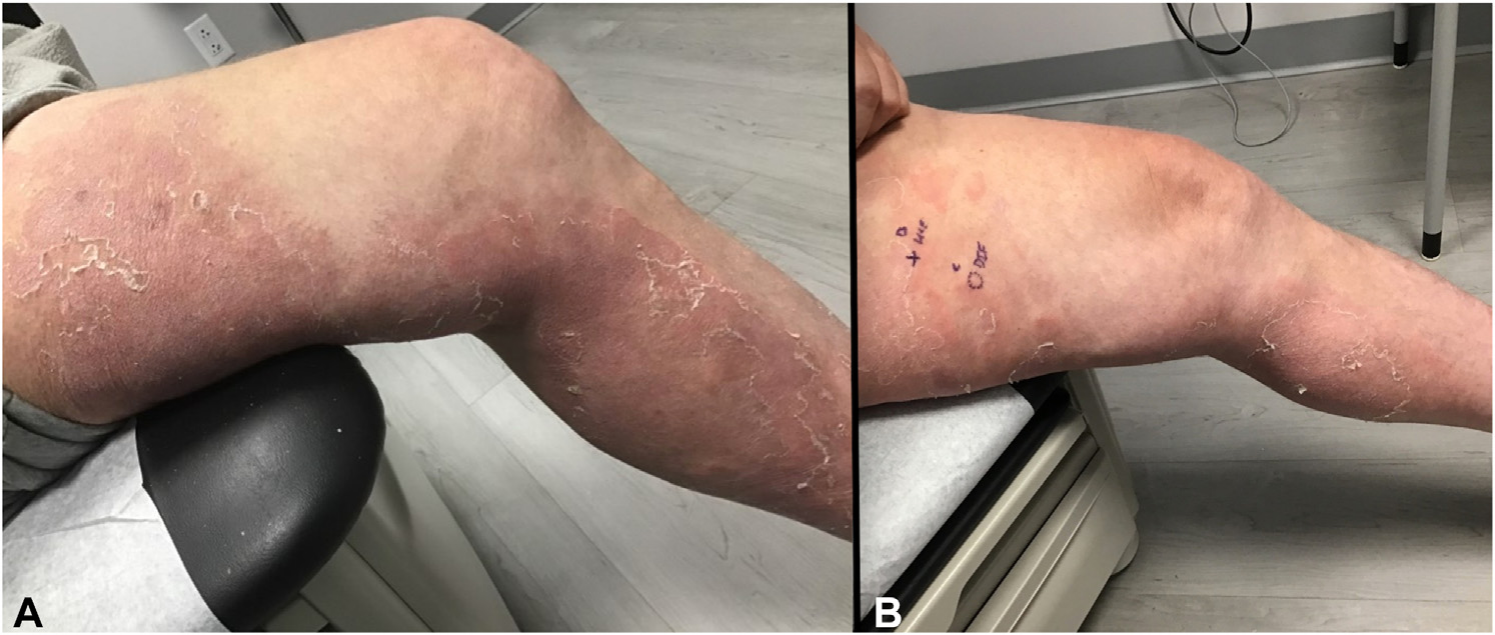
**A**, closer up view. **B**, Further away view, indicate where the biopsy was taken. Large, confluent, annular, *pink-red* erythematous patches, and thin plaques with fine scale at the periphery on the lower legs.

**Fig 2:**
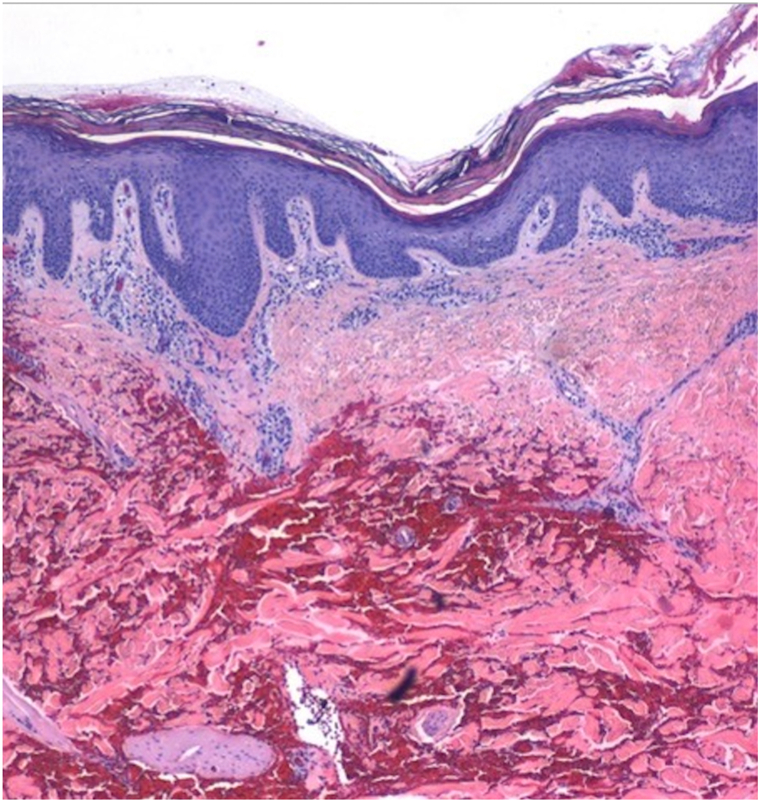
Serum crust, hyperkeratosis, irregular epidermal hyperplasia, and mild spongiosis seen on hematoxylin and eosin punch biopsy.

**Fig 3:**
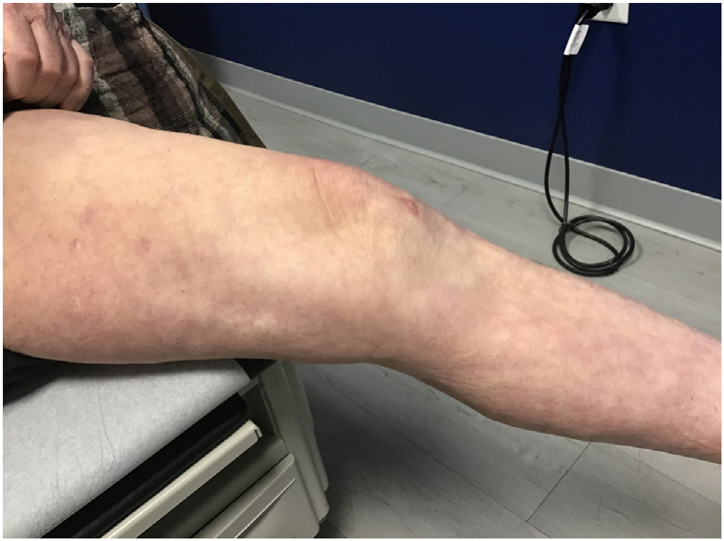
Complete clearing of annular lesions after 4 weeks of Rinvoq 15 mg orally once daily.

On examination, primarily on the lower legs, were large, confluent, annular, pink-red erythematous patches, and thin plaques with fine scale at the advancing edges. Various shades of red-brown patches were also present representing previous sites of involvement (Fig 1). The differential diagnoses included: eczematous dermatitis, psoriasis, pemphigus foliaceus, cutaneous T-cell lymphoma, id reaction, and EAC. Two hematoxylin and eosin punch biopsies showed serum crust, hyperkeratosis, irregular epidermal hyperplasia, mild spongiosis, and a superficial tight perivascular lymphocytic infiltrate around vessels (Fig 2). Direct immunofluorescence was negative. Previous treatments included mid- and high-potency corticosteroids, intramuscular triamcinolone, oral terbinafine, and dupilumab. Ultimately, the patient was started on upadacitinib 15 mg once daily and cleared completely after 4 weeks (Fig 3).

## Discussion

EAC was first described in 1881 as persistent, ring-shaped lesions with pruritus and was termed “erythema gyratum perstans”.^1^ The term “EAC” was introduced by Darier in 1916.^1^ EAC is most often an idiopathic eruption, initially presenting as single or multiple erythematous papules that form an annular plaque with central clearing. Superficial forms of EAC have scales on the advancing edge, which is commonly known as a “trailing scale”.^1^ In deep gyrate erythema, the advancing edges are elevated, and usually without associated scale or pruritus. Resolving lesions do not have residual scarring, but postinflammatory hyperpigmentation may be seen.^1^ These lesions can appear anywhere on the body but are more common on the trunk, proximal lower extremities, and the buttocks.^1^ EAC predominantly affects young or middle aged adults, but it can occur at any age.^1^

The pathogenesis of EAC is unknown but may be a type of reactive or delayed hypersensitivity reaction that can be triggered by a myriad of conditions. While the underlying cause is often unidentified, malignancy, medications, infections, systemic medical conditions, and pregnancy are known to precipitate EAC.^2^^,^^3^ When EAC is due to a malignancy, it is called paraneoplastic erythema centrifugum eruption. The most common underlying malignancies are lymphoproliferative disorders more so than solid tumors.^1^ Medications, including non-steroidal anti-inflammatory drugs, penicillins, antimalarials, spironolactone, hydrochlorothiazide, rituximab, and amitriptyline have all been associated with EAC.^4^ Infectious triggers are varied and include: bacterial, parasitic, dermatophyte, yeast, and viruses, most notably Epstein-Barr virus, poxvirus, HIV, and varicella.^1^ Systemic diseases such as Crohn’s disease, autoimmune endocrinopathies, and hypereosinophilic syndrome have also been associated.^1^ There have been three reports of EAC that were associated with pregnancy.^3^ When there is no definitive cause, the eruption is generally self-limited, yet it may persist for years or exhibit a seasonal presentation. If a definitive cause is identified, EAC typically resolves once the underlying cause is controlled.^1^

In this case, the diagnosis of EAC was favored for three reasons: (1) the clinical presentation, specifically the fine scale at the advancing edges; (2) the cyclical pattern of eruptions and the postinflammatory hyperpigmentation in sites of previous eruptions; and (3) failure to respond to high potency corticosteroids, intramuscular triamcinolone, and dupilumab.^1^ Although the pathology did not show textbook “coat-sleeve” lymphocytes, this finding is not always present and can be affected by both location and timing of biopsies.^1^^,^^5^ Eczematous dermatitis was the most likely competing diagnosis but lack of improvement with previous therapies argued against it. No other entities from our differential fit: features of psoriasis were not seen; pemphigus was not favored given the negative direct immunofluorescence and absence of erosions and crusting; and there were no atypical lymphocytes or epidermotropism making cutaneous T-cell lymphoma unlikely.

The treatment for EAC focuses on reducing the exanthem and relieving itch with topical steroids and antihistamines. Oral corticosteroids, antibiotics, and antifungals have also been used, but relapse is common.^1^ Previous case reports have listed topical calcipotriene, topical tacrolimus, narrowband ultraviolet B, subcutaneous etanercept, oral metronidazole, and subcutaneous interferon-alpha as potential therapies.^6^

Upadacitinib is a second-generation selective Janus kinase inhibitor (JAKi) that preferentially binds JAK1. It is 1 of a group of four tyrosine kinases (JAK1, JAK2, JAK3, and TYK2). It is Food and Drug Administration approved to treat atopic dermatitis, psoriatic arthritis, rheumatoid arthritis, ankylosing spondylitis, ulcerative colitis, Crohn’s disease and nonradiographic axial spondyloarthropathy.

EAC is a lymphohistiocytic inflammatory disorder mediated by T-cells. Upadacitinib successfully treats various inflammatory conditions that utilize the intracellular JAK/STAT pathway and interrupts signaling by multiple cytokines, including but likely not limited to, interferon-gamma, interleukin (IL)-4, IL-13, IL-22, IL-31 and thymic stromal lymphopoietin. Upadacitinib may improve symptoms of itch by reducing signaling of several of these cytokines like IL-4 and IL-13. It may reduce antigen presentation by decreasing the activity of thymic stromal lymphopoietin as well, which is a potent stimulator of antigen presenting cells. The T-cell mediated inflammation underlying EAC creates a rationale for using a JAKi as a potential therapy.

One weakness of this article is that this is a solitary case, and although not a common diagnosis of everyday clinical practice, the exact prevalence of EAC is difficult to define because most of the literature on EAC involves case reports and small reviews. Another potential weakness is that upadacitinib as used here represents an off-label indication, so acquisition of this therapy could prove challenging. Nonetheless, this case highlights an exciting new therapy with untapped potential. Currently, there are 324 clinical trials studying the use of JAKi. There are many diseases for which JAKi are already approved (atopic dermatitis, rheumatoid arthritis, psoriatic arthritis, inflammatory bowel disease) and many other diseases for which they are being investigated (psoriasis, hidradenitis suppurativa, systemic lupus erythematosus and many other conditions).^7^, ^8^, ^9^, ^10^ Therefore, dermatologists should become familiar with this class of medications and feel comfortable using these potentially very effective therapies.

## Conflicts of interest

Valerie Foy, DO: None. Meagen McCusker, MD: Abbvie (Consultant, Speaker). Michael Payette, MD, MBA: Abbvie (Consultant, Speaker).
